# Systematic review of Free and Cued Selective Reminding Test with Immediate Recall (FCSRT-IR) studies: normative data, clinical validity, and correlations with biomarkers

**DOI:** 10.1590/1980-5764-DN-2025-0399

**Published:** 2026-02-06

**Authors:** Priscila Mara Lorencini Selingardi, Clarisse Vasconcelos Friedlaender, Murilo Ricardo Zibetti, Gabriela Cabral Paciência, Paulo Caramelli, Mônica Sanches Yassuda

**Affiliations:** 1 Universidade de São Paulo, Faculdade de Medicina, Departamento de Neurologia, São Paulo SP, Brazil.; 2 Universidade Federal de Minas Gerais, Faculdade de Medicina, Unidade de Neurologia Cognitiva e do Comportamento, Belo Horizonte MG, Brazil.; 3 Universidade do Vale do Rio dos Sinos, Programa de Pós Graduação em Psicologia, São Leopoldo RS, Brazil.; 4 Universidade de São Paulo, Faculdade de Medicina, Programa de Gerontologia, São Paulo SP, Brazil.

**Keywords:** Alzheimer Disease, Biomarkers, Dementia, Memory, Doença de Alzheimer, Biomarcadores, Demência, Memória

## Abstract

The Free and Cued Selective Reminding Test with Immediate Recall (FCSRT-IR), developed by Grober and Buschke, is widely used to assess episodic memory and detect impairments, particularly in Alzheimer’s disease (AD). Recommended by the International Working Group (IWG) for early diagnosis, no prior review has synthesized its findings. This study reviewed articles on norms, clinical validity, and correlations with neuropathological biomarkers. Sixty-four studies were selected out of 728, covering normative data, clinical validation, and biomarker associations. The FCSRT-IR has normative data from seven countries and shows high diagnostic accuracy for mild cognitive impairment (MCI) and dementia, especially AD. In 20 studies, test scores significantly correlated with AD biomarkers. Thus, the FCSRT-IR supports early identification of episodic memory deficits, proving to be a valuable neuropsychological assessment tool.

## RESUMO.

O *Free and Cued Selective Reminding Test with Immediate Recall* (FCSRT-IR), desenvolvido por Grober e Buschke, é amplamente utilizado para avaliar a memória episódica e identificar prejuízos, especialmente na doença de Alzheimer (DA). Recomendado pelo International Working Group (IWG) para diagnóstico precoce, nenhuma revisão anterior havia sintetizado seus achados. Este estudo analisou artigos sobre normas, validade clínica e correlações com biomarcadores neuropatológicos. Foram selecionados 64 estudos do total de 728, abrangendo dados normativos, validação clínica e associações com biomarcadores. O FCSRT-IR possui normas em sete países e demonstra alta acurácia para comprometimento cognitivo leve (CCL) e demência, especialmente DA. Em 20 estudos, os escores apresentaram correlação significativa com biomarcadores da doença. Assim, o FCSRT-IR contribui para a identificação precoce de déficits na memória episódica, sendo uma ferramenta valiosa na avaliação neuropsicológica.

## INTRODUCTION

 Fifty million people worldwide are affected by some form of dementia, AD being the main etiology, accounting for approximately 60–70% of all dementia cases^1^ . To improve the care and management of patients with dementia, researchers have focused on identifying strategies for diagnosing this condition as early as possible^2^ . Neuropsychological assessment, and more specifically episodic memory assessment, plays a prominent role in the diagnostic process, especially when AD is the main diagnostic hypothesis. Memory assessment is also vital for the identification of mild cognitive impairment (MCI) as it poses an increased risk of conversion to dementia^3^ . 

 Several validated tools are currently available for assessing episodic memory, involving the encoding of new information such as short stories^4^ , word lists^5,6^, and visual stimuli such as black-and-white or colored figures^7,8^. The Free and Cued Selective Reminding Test (FCSRT)^9^ requires the memorization of 16 words (word version) or 16 black and white pictures (picture version) based on semantic cues. Initially, the test was called Selective Reminding Test (SRT), and its main characteristic was the repeated presentation of items that were not recalled in consecutive free recall trials. In 1984, the test was improved, and the new version included the provision of semantic cues during recall trials for information that was not spontaneously recalled^10^ . 

**Figure 1:**
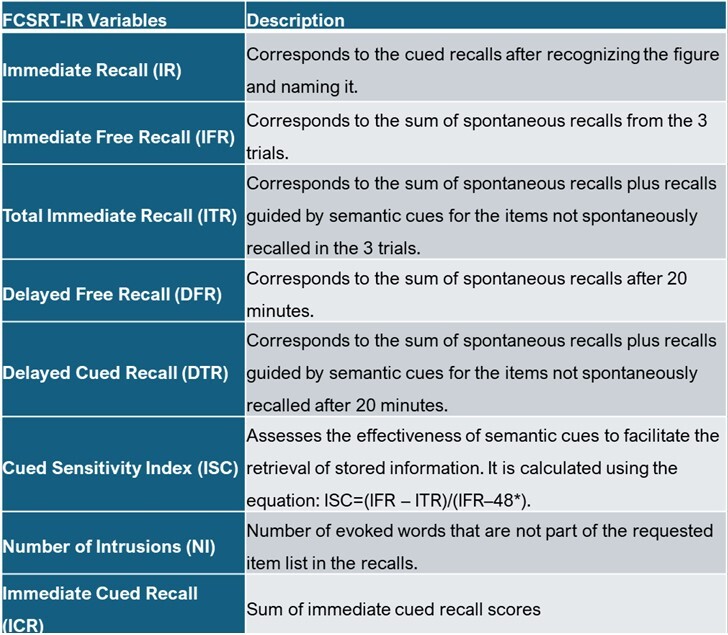
Descriptions of Free and Cued Selective Reminding Test with Immediate Recall (FCSRT-IR) variables.

 In 1987, an immediate cued recall was added, and the test was renamed of Free and Cued Selective Reminding Test with Immediate Recall (FCSRT-IR)^9^ . In this revised version, two independent encoding phases based on semantic cues were introduced. In the first phase, items are presented in groups of four, and the participant is instructed to identify the word or picture (among four alternatives) that corresponds to a semantic category cue (e.g., fruit) provided by the examiner. After the four items are correctly identified through naming, the card is removed and the immediate recall (IR) phase begins, supported by categorical cues. If the participant is unable to recall an item in response to its cue, the card is re-presented, providing another opportunity for immediate cued recall. This procedure is successively applied to the remaining sets, in groups of four, until all 16 stimuli are learned. Next, there are three consecutive free recall trials, and the Immediate Free Recall (IFR) corresponds to their sum. In each, semantic cues are offered for items that are not recalled. The Immediate Total Recall (ITR) reflects the sum of spontaneously recalled items and those remembered with cues. Finally, after 20 minutes, the person is asked to freely recall all items (Delayed Free Recall - DFR), and semantic cues are offered for the forgotten ones (Delayed Total Recall - DTR - spontaneous plus cued recall). The variables assessed by the FCSRT-IR and their respective descriptions are summarized in Figure 1. 

 The FCSRT-IR is different from the usual episodic memory tests, as the two independent encoding phases are dependent upon the presentation of semantic cues by the examiner. This feature may aid in the identification of patients with AD, who tend to have memory consolidation deficits, from other conditions in which memory impairment occurs to attentional or executive function deficits^11^ . 

 More recently, two additional parameters derived from FCSRT-IR have been shown to be particularly useful in staging changes in episodic memory. Objective Memory Impairment (SOMI) classifies memory impairment into five stages that may precede dementia. This classification system, which ranges from 0 to 4, evaluates the stages of memory impairment based on IFR and ITR scores. Individuals with impairment only in IFR receive a score between 1 and 2, whereas those with impairment in both IFR and ITR (suggesting that they no longer benefit from cues) receive scores of 3 or 4, indicating storage impairment^12^ . The Index of Sensitivity of Cueing (ISC)^10,13^ assesses the effectiveness of cues in information retrieval and is measured by the ratio of the difference between recall with and without cues relative to the total number of items recalled without cues. It varies from 0 to 1, with higher values indicating greater benefits from cues. 

 To the best of our knowledge, no systematic review has yet consolidated studies on FCSRT-IR concerning normative data, clinical validation, and correlations with biomarkers. Therefore, this review aimed to identify and analyze studies on FCSRT-IR to offer an overview of the current use of the test and identify normative and clinical validation studies, including those that correlated the test with AD biomarkers. 

## METHODS

 The present review was conducted following the majority of the recommendations described in the Preferred Reporting Items for Systematic Reviews and Meta-Analyses (PRISMA) guidelines (http://www.prisma-statement.org), with the following exceptions: the quality of the studies was not analyzed due to time and human resource limitations, and the study was not registered in the International Prospective Register of Systematic Reviews (PROSPERO). Alternatively, this review was registered in the Open Science Framework (OSF) (DOI 10.17605/OSF.IO/YPH47). 

### Literature search

 On July 9, 2023, a search was conducted in five databases — United States National Library of Medicine (PubMed), Cochrane, Medical Literature Analysis and Retrieval System Online (Medline), Scientific Electronic Library Online (SciELO), and Latin American and Caribbean Health Sciences Literature (LILACS) — using the following search terms: (Free and Cued Selective Reminding Test) OR (Buschke test) OR (Buschke memory test) OR (enhanced cued recall) AND (norms) OR (normative data) OR (standardization) OR (validation). An update was made on September 23, 2024. 

### Objective

 The objective of this review was to identify and analyze previously published studies on FCSRT-IR, including test norms, clinical validation, and correlation with biomarkers. 

### Study selection

 After the articles were identified, they were screened using the Rayyan software. First, all duplicates were removed, and the titles and abstracts were analyzed by two independent reviewers (PMLS and GCP) to select studies. Discrepancies were resolved by a third reviewer (MSY). The following inclusion and exclusion criteria were adopted: studies investigating FCSRT-IR, normative studies, clinical validation studies, and studies correlating the test with biomarkers (structural, functional, and molecular neuroimaging exams). Review studies, studies with samples of children and adolescents, studies with different versions of the test, and studies in languages other than English, Spanish, or Portuguese were excluded. 

### Data extraction and analysis

 The following data were extracted: first author and year of publication, country of origin, objectives, study design, sample size and characteristics, version of the FCSRT-IR (words or pictures), FCSRT-IR variables analyzed, and main findings. For studies on clinical validation and biomarkers, information regarding diagnostic accuracy and the type of biomarker used was also collected, as shown in Supplementary Material Tables S1, S2, and S3 (available at  https://www.demneuropsy.org/wp-content/uploads/2025/11/DN-2025.0399-Supplementary-Material.docx). The extracted data were verified by an additional reviewer (MSY). 

## RESULTS

**Figure 2:**
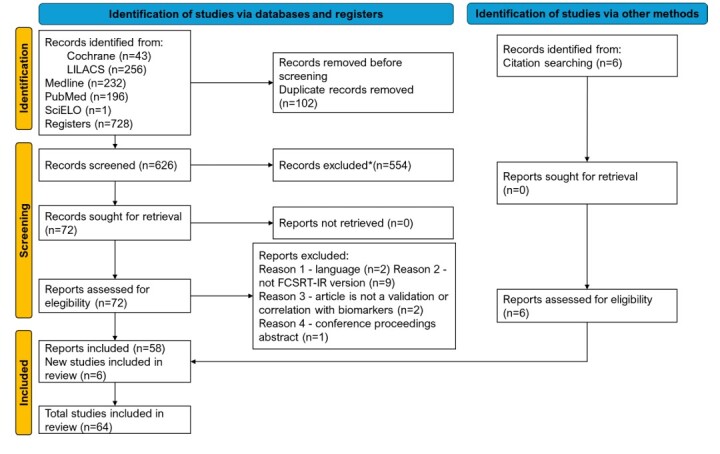
Preferred Reporting Items for Systematic Reviews and Meta-Analyses (PRISMA) flowchart.

 The PRISMA flowchart (Figure 2) illustrates the article selection process. Our search strategy revealed 728 articles, which were distributed as follows: 43 from Cochrane, 256 from LILACS, 232 from Medline, 196 from PubMed, and one from SciELO. After removing duplicates (n=102), 626 abstracts were screened and 72 articles were selected and read in full. Of these, 14 were excluded because they did not meet the inclusion criteria, while six others were included after being identified during the full-text review. Thus, 64 articles remained in the review: 16 normative studies (Table S1), 27 clinical validation studies (Table S2), and 24 studies correlating FCSRT-IR with biomarkers (Table S3). Three articles were included in more than one table, because they investigated more than one topic^14-16^ . 

### Normative studies

 Sixteen normative studies from seven countries were identified: Argentina^17^ , Denmark^18^ , France^19-21^, Italy^10,13^, Mexico^22^ , and Spain^23-28^ USA^15 ,29^ (Table S1). Except for two studies^21,29^, all included cross-sectional analyses. One of them^29^ followed participants over time to exclude those who developed cognitive impairment from norms. With only two exceptions^13,15^, all normative studies used the word version of the FCSRT-IR. Additionally, three studies included a recognition phase in the word version of the instrument^17,19 ,21^, whereas another focused on developing normative data for a recognition task in the FCSRT-IR^25^ . The study sample consisted of participants aged 18–94 years. Most studies have indicated that age and/or education influence performance on the FCSRT-IR^13,15 ,17,18 ,20-23 ,26,28^. However, two studies observed the influence of education but not of age^24,27^. Three studies reported the influence of sex, with a better performance in women^20,26 ,27^. 

### Clinical validation studies

 Twenty-seven studies investigated the clinical validity of the FCSRT-IR (Table S2); 11 used only the picture version,^15,16,30 -38^ and 15 used only the word version^2,14,39-51^. One study used both versions to compare results^52^ . 

 Most clinical studies were conducted in the USA^15,16,30,32 ,35,39 ,41^ and Europe (seven from France^14,40 ,42,45 ,47-49^, four from Portugal^44,46,50 ,53^, three from Italy^34,37 ,38^, and one from Spain^2^ ). Additionally, in Latin America, such as Chile^36,52^, Peru^33^ , Brazil^31^ , and Taiwan^51^ , this instrument has also been investigated. 

 Clinical studies have evaluated memory performance in individuals diagnosed with MCI^33,36,40,44,46 ,47,51 ,53^, AD,^2,14,16,30 ,31,33 -35,37 -40,42 ,44-47 ,49-54^ or other dementias^14,34,39 ,50,51 ,53^, the diagnostic accuracy of the test for identifying AD^30,31,33 ,35,38 ,40,42 ,44-47 ,52^, and dementia in general^15,36 ,41,48^. Furthermore, the studies investigated the contribution of the test to the differential diagnosis among various types of dementia^2,34,37,49 ,50^ and its ability to differentiate MCI from AD ^16,33,36 ,46,53^. The risk of developing MCI^32,36^ or dementia^35,38 ,42,44 ,45^ has also been studied using the FCSRT-IR. 

 Studies have reported cut-off scores for different measures extracted from the FCSRTIR^30,31,33,35,38 ,40-42 ,44,46 ,48,52 ,53^. Sensitivity has been estimated to range from 54 to 98% and specificity from 39 to 100%.^30,31 ,35,36 ,38,40 -42,45 ,46,48 ,52,53^ Some of these studies have reported Area Under the Curve (AUC)^16,31 ,36,40 ,49,52^ values ranging from 64 to 99%, as well as Positive Predictive Values (PPV), Negative Predictive Values (NPV)^38,42,45 ,46,48 ,53^, and Hazard Ratios for the development of dementia^55^ . These rates tend to vary depending on the context in which the study was conducted, sample characteristics, objectives, or the specific FCSRT-IR measure analyzed. Diagnostic accuracy tends to increase when samples include patients diagnosed with AD and the sensitivity and specificity rates are higher for the IFR and DTR measures. 

 Five studies investigated the diagnostic accuracy of ISC^35-38 ,40^ and SOMI scores^16^ . One of them suggested that the ISC is the most accurate measure for detecting AD compared to other variables derived from the instrument^40^ . Additionally, one study indicated that patients with vascular dementia and AD had lower ISC values in immediate trials than controls and patients with frontotemporal dementia (FTD)^37^ . Findings indicated that participants classified as SOMI 4 or with even more impaired performance had a significantly increased likelihood of exhibiting AD-positive neuropathology^16^ . 

### Studies reporting correlations with biomarkers

 All 24 studies reported correlations between FCSRT-IR and neuropathological biomarkers, genetic markers, and glucose tolerance. These studies were conducted in Europe^14,56-59^, USA^16,32 ,55,70 -74^, and Chile^75^. 

 The samples in these studies were composed of healthy participants and patients with MCI^12,57,60-64,73 ,75^, FTD^14,69^, AD^14,16 ,55,59 ,71,74^ Cortical Basal Syndrome (CBS), or Progressive Supranuclear Palsy (PSP)^58^ ; carriers of the e4 allele;^70^ patients who had had a stroke;^56^ or those with alterations in glucose tolerance^66^ . 

 Studies using structural Magnetic Resonance Imaging (MRI) have shown a significant association between lower IFR and ITR scores of the word version of the FCSRT-IR and a higher number of intrusions^56,58,69^ with medial temporal lobe atrophy, particularly in the hippocampus^60 ,63,66 ,69,70 ,73,74^. Notably, one of these studies reported that individuals with elevated 1-hour postload glucose levels (NGT1-h-high) showed significantly reduced hippocampal volumes and lower right hippocampal diffusivity, in addition to poorer performance, specifically on the DTR^66^ . 

 Two studies used CT scanning techniques to examine the associations between FCSRT-IR scores and changes in brain areas. The results indicated that changes in the medial temporal lobe (MTL) were associated with lower scores on memory measures, such as ITR and IFR, compared with individuals without MTL atrophy. Moreover, the percentage of benefit from cues was lower and the number of intrusions was higher in the MTL atrophy group^56,69^. 

 Two other studies using MRI compared word and picture versions to investigate the brain areas involved in the performance of each test version^63,75^. One study examined the correlation between the two versions of cortical atrophy in patients with MCI^63^ . The results indicated an association between the IFR score of the word version and a reduction in right hippocampal volume, while in the picture version this score was correlated with changes in the volume of the fusiform gyrus and visual cortex. Regarding the ITR and ISC scores, in the word version, there was a correlation with atrophy in both the hippocampi. In contrast, in the picture version, ITR was associated with atrophy of the left and right fusiform gyri, and ISC was associated only with atrophy of the left fusiform gyrus^63^ . Another study explored the association of the two versions with atrophy of the hippocampus and other cortical structures in patients with mild AD and in cognitively normal controls^75^ . A correlation was found between the IFR of the word version and atrophy of the right middle frontal gyrus and between the picture version and atrophy of the right temporal fusiform gyrus and bilateral parahippocampal regions^75^ . Thus, both studies agree that the scores of the picture version of the FCSRT-IR are associated with atrophy in the fusiform gyrus, visual cortex, and parahippocampal region^63,75^. An additional study indicated that IFR and DFR are associated with the left hippocampal volume^74^ . 

 Other studies using MRI have identified an association between IFR^56,73 ,74^, ITR^56,60,70^ or DFR^56,60 ,66,67 ,74^ deficits in the word version of the FCSRT-IR and left thalamic volume alterations^56^ , as well as atrophy in the hippocampus^60,66,67 ,71,73 ,74^ and right middle frontal gyrus^75^ . In the picture version, IFR was associated with atrophy of the right temporal fusiform gyrus^63,75^ and parahippocampal region^75^ . Additionally, a reduced cueing benefit was associated with atrophy of the posterior cingulate gyrus, precuneus^62^ , and frontal lesions^56^ . 

 One functional neuroimaging study using functional MRI (fMRI) suggested that poorer performance in IFR was correlated with fMRI using the blood-oxygen-level-dependent signal (fMRI-BOLD) signal changes in the pre- and post-central gyri, inferior parietal lobe, left precuneus, right middle frontal gyrus, left and right hippocampi, and parahippocampus^71^ . 

 A study using single Photon Emission Computed Tomography (SPECT) observed distinct patterns in ITR between patients with FTD and AD, indicating differences in retrieval strategies. Specifically, FTD patients benefited more from cues than did AD patients^14^ . Moreover, another study reported that measures such as IFR and ITR combined with SPECT imaging may enhance the diagnostic accuracy for AD^57^ . 

 Regarding positron emission tomography-fluorodeoxyglucose (PET-FDG) scans, one study indicated that patients with AD performed worse on the IFR than controls, and this score was associated with reduced metabolism in the right superior, inferior frontal gyri, parahippocampal, and frontal areas^59^ . 

 Two studies used amyloid PET to compare the amyloid burden with the performance in the FCSRT-IR.^12,64^ One study found that lower scores on ITR and ISC were negatively correlated with amyloid burden in the precuneus and posterior cingulate cortex, especially in patients under 75 years of age^64^ . Another study identified that higher stages of SOMI were associated with higher amyloid pathology burden and smaller volumes of the hippocampus, entorhinal cortex, and lower temporal lobes. 

 Additionally, examination of molecular biomarkers for AD, both in cerebrospinal fluid (CSF)^58,61,68^ and blood samples^65,67,70^, indicated that ITR scores showed a stronger correlation with AD biomarkers compared to other memory tests^61^ , exhibiting high sensitivity for detecting this pathology, even in its prodromal stages^58,65,68,70^. Regarding the histological examination, performance on the IFR declined progressively from Braak stages III and IV, whereas ITR showed a significant decline only from stage IV to VI^55^ . This result suggests that IFR is more sensitive to early changes and that changes in cue-based performance only decline in the more advanced stages of the disease. 

 One study reported a strong correlation between lower ISC and IFR scores, and the likelihood of amyloid PET positivity. An ISC score below 0.5 revealed a probability of over 85% for amyloid positivity in patients under 76 years of age^64^ . The predictive ability of the ISC was enhanced when the examination focused on specific brain areas, such as the precuneus and posterior cingulate cortex, rather than considering the global amyloid load^64^ . 

 In a study using magnetoencephalography (MEG), lower alpha frequencies in the temporo-occipital regions were associated with lower ISC scores^72^ . Patients showed lower rates of encoding and long-term retention than healthy older adults. These findings suggest that memory dysfunction is associated with decreased alpha activity and pathological aging. 

 Finally, SOMI scores were shown to be strong predictors of AD pathology and Braak stages^16^ . Additionally, a higher SOMI was associated with a greater amyloid burden and reduced volume of the hippocampus and entorhinal cortex^12^ . 

## DISCUSSION

 This review aimed to identify and analyze studies focusing on FCSRT-IR to provide an overview of the current scientific evidence regarding this important diagnostic tool. The search yielded 728 articles, 64 of which were selected for review. Among them, 16 pertained to norms, 27 to clinical validity, and 24 to biomarker correlation studies. 

 Normative studies were conducted almost exclusively using the word version of FCSRT-IR. Therefore, we identified an important gap in the availability of norms for the picture version of the test that may be particularly relevant for assessing individuals with lower education. In addition, regarding the clinical relevance of the normative data, the results indicated that the test scores are influenced by age and/or education^13,15,17,18,20 -23,26 ,28^, some of them pointing just to the influence of education^24,27^. These findings align with previous literature suggesting that performance on the FCSRT-IR should be interpreted in the context of an individual’s sociodemographic characteristics, as lower scores may result from lower education levels in older age groups^76^ . Therefore, for diagnostic purposes, the availability of norms stratified by age and education is crucial. We also highlight that three studies suggested that the test may be influenced by sex. 

 Studies have reported that performance on the picture version of the FRSRT-IR is usually higher than that on the word version^52 ,77^. Several factors could explain this finding. According to dual encoding theory, images are easier to store because of dual mental representation (verbal and visual), while words are stored only verbally^35,78^. Additionally, pictures might engage a broader brain network in recognizing object categories than words. This may enhance memorization, as indicated by the results of imaging analyses^75^ . 

 The high accuracy of FCSRT-IR in identifying AD patients can be attributed to its ability to reveal underlying hippocampal-related memory consolidation deficits^79^ . Even in the early stages of the disease, patients show marked impairments in encoding new information in both free and cued recall^2 ,14,34 ,37,50^. This pattern of performance, in which patients with AD are unable to benefit from retrieval cues, contrasts significantly with other dementias when cued recall often improves performance. Therefore, the structured approach of the FCSRT-IR for encoding and retrieval may explain its higher diagnostic precision compared with other memory tests. 

 Some studies have highlighted the utility of FCSRT-IR in identifying MCI^33^ and differentiating between MCI and AD^46,53^, as well as distinguishing between healthy controls and AD^36^ . The free recall measures (IFR and DFR) of FCSRT-IR were particularly effective in identifying the risk of developing MCI^32^ . Its effectiveness in differentiating MCI from AD remains unclear, highlighting the need for further research to address this issue. 

 FCSRT-IR has also proven useful for identifying dementia in Parkinson’s disease^48^ and for assessing the risk of cognitive impairment and dementia in patients with cardiovascular disease^42^ . FCSRT-IR appears to be effective in distinguishing AD from other dementias, with less prominent memory impairment, as it may assist in the differentiation of AD from subcortical vascular dementia^34,37^ or FTD^14,37,50,51 ,69^. These studies demonstrated that patients with AD exhibit a reduced ability to retain learned information over time, showing lower DTR scores. They benefited less from cues and experienced more intrusions than patients with vascular cognitive impairment or FTD. These findings suggested that AD-related memory deficits are associated with encoding and storage deficits^37^ . This distinction is supported by the fact that neurodegeneration is more prominent in the medial-temporal region^42^ in AD. 

 The reviewed studies have suggested the validity of this tool as a clinical marker for neuropathological changes associated with neurodegenerative diseases. Specifically, atrophy in the medial temporal lobe, particularly in the hippocampus, as well as the presence of pathological tau and amyloid and tau biomarkers characteristic of AD, have shown a strong correlation with impairments in IFR and ITR measures^12,56,60,62,63 ,66,67 ,69,70 ,73-75^ . 

 Neuropathological changes in the medial temporal lobe appeared to impair the ability to benefit from cues, as assessed by the ISC score^56-61,69-71 ,73,74^. Additionally, lower ISC scores have been associated with reduced alpha frequencies in the temporo-occipital regions^72^ with amyloid pathology, especially in patients younger than 76 years^64^ . 

 Two studies compared the word and picture versions of the FCSRT-IR scores with AD biomarkers^63,75^. No significant differences were found in the ability to identify biomarker changes between the two test versions. 

 Higher SOMI has been linked to a greater amyloid pathology burden and reduced volume in key brain areas for memory^12^ . SOMI scores were positively correlated with Braak stages, while more advanced stages were associated with decreased cued recall. In summary, SOMI and ISC scores are useful tools for tracking the progression of memory impairment. Such scores may assist in decisions regarding intervention strategies for AD and disease management. 

 In conclusion, the results of this review reinforce the validity and usefulness of FCSRT-IR in assessing memory impairment, particularly within the AD spectrum. These studies emphasized the importance of normative data, as test scores are significantly influenced by sociodemographic factors. Research conducted in various countries has further highlighted the clinical validity of this tool in identifying dementia syndromes with episodic memory impairment, including their preclinical stages. Studies using biomarkers have also reinforced the clinical validity of FCSRT-IR. 

 These findings suggest that the use of this instrument in clinical settings can enhance early diagnosis of AD and assist in the implementation of interventions. Moreover, FCSRT-IR can play a pivotal role in the differential diagnosis between AD and other dementias, with memory difficulties due to attention deficits or executive function impairments. 

 Future research should continue to investigate the relationship between neuropathological biomarkers and performance on the FCSRT-IR to gain a deeper understanding of the mechanisms underlying memory impairment in dementia. The need for normative studies on FCSRT-IR, particularly in Latin American countries using its picture version, is also highlighted, as dementia cases are often underdiagnosed in this region^80^ . 

## Data Availability

No new data were generated or analyzed in this study.
