# Supplementary material for: Systematic review of Free and Cued Selective Reminding Test with Immediate Recall (FCSRT-IR) studies: normative data, clinical validity, and correlations with biomarkers
Source: Dement Neuropsychol. 2026 Feb 6;20:e20250399. doi: 10.1590/1980-5764-DN-2025-0399 (PMC12885474; doi:10.1590/1980-5764-DN-2025-0399)
Supplement: Supplementary Material [file 1980-5764-dn-20-e20250399-md3.docx]

**Supplementary Material**

**Table S1.** Normative studies of the FCSRT

| **Author, year** | **Country** | **Study design** | **Objective** | **Version** | **Sample’s Characteristics** | **Variables**  **FCSRT** | **Results** |
| --- | --- | --- | --- | --- | --- | --- | --- |
| Labos et al., 2008 | Argentina | Cross-sectional | Develop norms for the Spanish version of the FCSRT-IR for the Argentine population. | Words | **Total sample (n= 239)**  Age(years): 56.4; ±17.1 (range 20 **-** 70)  Education (years): 12.03; ±4.25 (<8 y = 62; ≥8 = 177) | IR; IFR; ICR; DFR; DTR, Recognition | The ability to learn verbal stimuli decreases with age, especially after 60, with a more significant decline after 70, while recognition remains stable. Educational level improves learning ability. There are no gender differences in memory performance, and semantic cues help recover almost 100% of the stimuli in all age groups except in the oldest old (90.01%). |
| Holtzer et al., 2009 | USA | Cross-sectional, Longitudinal  (10 years) | Develop robust norms for individuals aged 70 and older on various neuropsychological tests including the FCSRT-IR. | Words | **Total sample (n=1251)**  Age (years): 78.7; ±5.16  Education (years): 12.7; ±3.9  Robust sample (n = 307)  Age (years): 77.2; ±4.47 (70-89)  Education (years).: 13.5; 3.46 (2-24)  Incident Dementia (n= 58)  Age (years): 81.4; ±12.5 (72-95)  Education (years).: 12.5; ±3.79 (0-22)  Prevalent Dementia (n= 75)  Age (years): 82.6; ±5.49 (70-100)  Education (years)..: 106; ±4.3 (2-25)  Lost to follow-up (n = 314)  Age (years): 78.4; ±5.14  Education (years).: 11.9; ± 3.58 | IFR; ITR. | Robust norms more accurately reflect cognitive functioning in normal aging than traditional cross-sectional norms which may include participants with pre-clinical dementia. |
| Peña-Casanova et al., 2009 | Spain | Cross-sectional | Developing norms for the FCSRT-IR for the Spanish population aged 50 and above. | Words | **Total sample (n = 340)**Age (years): (50 – 94)  Education (years).: (≤ 5 to ≥16) | IFR (1st trial); IFR; ITR; DFR; DTR; Retention (Delayed Total Recall/Total 3rd Trial Score) | Age and education influenced the FCSRT-IR scores, although the years of education had a mild effect. Sex had a irrelevant effect. |
| Frasson et al., 2011 | Italy | Cross-sectional | Provide normative data for the Italian version of the FCSRT-IR. | Colored Pictures  (12 items) | **Total sample (n = 227)**  Age (years): 66.6; ±13.15 (40 – 94)  Education (years).:±11.1; 4.58 (4 – 21) | IFR; ITR; DFR; DTR; ISC, intrusions | Age significantly influenced ITR and DFR, while education and sex only influenced IFR, with better performance for women with higher education. |
| Mokri et al., 2013 | Mexico | Cross-sectional | Provide normative data for various cognitive tests including the Mexican version of the FCSRT. | Words | **Total sample (n = 538)**  Age (years): >70 years  Education (years): 0 - 6 years | IFR; ITR; DFR; DTR. | The measures of IFR, ITR, DFR and DTR were significanly influenced by age and education. Gender influenced the DFR, although the effect was not as pronounced as that of age or education. |
| Palomo et al., 2013 | Spain | Cross-sectional | Provide normative data for the FCSRT-IR and ROCF for young adults. | Words | **Total sample (n = 179)**  Age range: 18 – 49  Education range:3 - 19 | IFR (1^st^ trial); IFR.; ITR; DFR; DTR. | Results indicate the influence of years of education on IFR and ITR. Age and gender did not influence the FCSRT-IR measures. |
| Dion et al., 2015 | France | Cross-sectional | Provide normative data for the French version of the FCSRT-IR for the population of Quebec according to age, education level, and sex. | Words | **Total sample (n = 566)**  Age (years): 72.8;±7.3 (50 - 88)  Education (years): 13.3; ±4 (3 - 23) | IFR (each one of the 3 trails) IFR; DTR, Recognition | Age was only related to recognition performance, while education positively impacted both IFR and ITR scores. Women outperformed men on the first trial of IFR and DFR. |
| Girtler et al., 2015 | Italy | Cross-sectional | Provide normative data for the Italian version of the FCSRT-IR according to age, education level, and sex. | Words | **Total sample (n = 194)**  Age (years): 57.73; ±19.57 (20 - 90)  Education (years): (3 - +13) | IFR; ITR; DFR; DTR; ISC (ITR-IFR)/(48-IFR), number of intrusions | Age and education influenced performance in both IFR, ITR, DFR and DTR. |
| Grober et al., 2015 | USA | Cross-sectional | Develop robust norms for the FCSRT-IR picture version and compare them with conventional norms to identifyg incident dementia. | Pictures | **Total sample (n = 836)**  Age (years): 76.8; ± 4.7 (70 a 89)  Education (years): 14.3; ± 3.1 (3 a 13)  **Grups:**  **Conventional (n = 418)**  Age (years): 78.2; ± 4.7  Education (years): 14.1; ± 3.2  **Robust (n = 340)**  Age (years): 77.4; ± 4.5  Education (years): 14.4; ± 3.0  **Incident Dementia (n = 78)**  Age (years): 81.6; ± 4.4  Education (years): 13.7; ± 2.8 | IFR | There was a negative correlation with age and a positive one with education. |
| Vogel et al., 2018 | Denmark | Cross-sectional | Develop norms for various cognitive tests including the FCSRT-IR for the Danish population according to age, sex, and years of education. | Words | **Total sample (n = 131)**  Age: 72.9; ±8.1 (60 - 96)  Education (years): 12.9; ±2.8 (8-17) | IFR; ITR; DFR; DTR. | Age had a significant impact on both IFR and DFR of the FCSRT. |
| Ouvrard et al., 2019 | France | Cross-sectional | Provide normative data for the French version of the FCSRT- IR and other cognitive tests. | Words | **Total sample (n = 51.879)**  Age (years): 57.7; ±7.1 (20 - 90)  Education (years): 3,4% - <5years; 49% - 5 -13years; 47,4% - >= 14 years | IFR; ITR; DFR; DTR. | Age and education slightly influenced the performance of all FCSRT measures, while gender only had an effect on individuals with less education, with women performing better. |
| Bonete-López et al., 2021 | Spain | Cross-sectional | Develop norms for the recognition task of the Spanish version of the FCSRT-IR. | Words  (Recognition task – 40 items) | **Total sample (n = 96)**  Age (years): 65.71; ±6.68 (55-87)  Education (years): 11.39; ±3.37 (3 - 19) | - | The results indicated high accuracy in differentiating between correct and incorrect items even in older, cognitively healthy adults with near-ceiling performance on hits and near-floor performance on false recognitions. |
| Brugulat-Serrat et al., 2021 | Spain | Cross-sectional | Develop more robust norms for the FCSRT and WMS-IV ML for subjects with normal levels of amyloid and tau biomarkers in the CSF. | Words | **Total sample (n = 248)**  Age (years): 60.5; ±4.5 (50-70)  Education (years): 13.6; ±3.5 (8-20) | IFR; ITR; DFR; DTR. | IFR, ITR and DFR measures were significantly affected by sex. Education had a significant effect all measures (IFR, ITR, DFR, DTR), while age only affected the DFR. |
| Calderón-Rúbio et al., 2021 | Spain | Cross-sectional | Develop norms for cognitive tests including the FCSRT for the Spanish population aged over 55 years. | Words | **Total sample (n = 103)**  Age (years): 65.7; ±6.56 (55 - 87)  Education (years): 11.44; ±3.46 (3 -22) | IFR; DTR. | No effect of age was found, but sex had a significant impact on ITR and DTR, with women outperforming men. Education also showed a significant effect on ITR and DTR, with higher education generally correlating with better performance on these measures. When the ITR is considered, the effect of education on DTR disappears. |
| Grau-Guinea et al., 2021 | Spain | Cross-sectional | Create norms for an alternative version to the Spanish version of the FCSRT. | Words | **Total sample (n = 232)**  Age (years): 55.3; ±19.2 (18 – 90)  Education (years):11.5; ±4.27 | IFR(1st trial); IFR; ITR; DFR; DTR; | Although version B correlated with age and level of education, there were significant differences IFR scores between the two versions, with version A showing higher scores. ITR and DTR for both versions, were considered equivalent. |
| Ceresetti et al., 2023 | France | Longtudinal  (20 years) | To present normative data from cognitive tests, including the FCSRT-IR, over the follow-up period, with a focus on fatal and non-fatal cardiovascular and cerebrovascular events. | Words | **62 -69 years old (n=929)**  Age (years): 65.63; ±0.81  Education: no diplom = 8%; CEP=43%, CAP= 29%, BAC and + = 20%  **71 -78 years old (n=631)**  Age (years): 74.75; ±0.99  Education: no diplom = 8%; CEP=38%, CAP= 34%, BAC and + = 20%  **81 88 years old (n=293)**  Age (years): 84.67; ±1.33  Education: no diplom = 8%; CEP=34%, CAP= 37%, BAC and + = 21% | IFR, ITR, DFR, DTR, Recognition, Intrusions, Semantically related intrusions, Unrelated intrusions | The performance on the FCSRT was positively influenced by education and negatively influenced by age. Women performed better in all variables except for recognition. |

Abbreviations: AD, Alzheimer's Disease; BAC, baccalaureate (high school); CAP= certificate of professional aptitude; CEP= primary school certificate; DFR, Delayed Free Recall; DTR, Delayed Total Recall; IFR, Immediate Free Recall; ITR, Immediate Total Recall; ISC, Index Sensitivity of Cued; LM, Logical Memory; aMCI, Amnestic Mild Cognitive Impairment; mdMCI, Mild Cognitive Impairment Multiple Domains; naMCI, No Amnestic Mild Cognitive Impairment; ROCF, Rey-Osterrieth Complex Figura; SABIEX, Spanish Bioinformatics Institute for Experimentation; SCD, Subjective Cognitive Decline.

**Table S2.** Clinical validity studies of the FCSRT-IR

| **Author, year** | **Country** | **Study Design**  **(follow-up time)** | **Objective** | **Version** | **Sample Characteristics** | **FCSRT Variables studied** | **Diagnostic Accuracy**  **(cutoff, Se, Sp, AUC)** | **Results** |
| --- | --- | --- | --- | --- | --- | --- | --- | --- |
| Grober e Kawas, 1997 | USA | Longitudinal  (7 years) me parece que os resultados se referem a comparações cross-sectional | To investigate whether the retention deficit occurs before or after the learning deficit in participants who later developed AD, using FCSRT-IR | Words | **Total sample (n= 80)**  **Control** (n = 60)  Age (years): 79.0; ±5.1  Education (years): 17.0; ±2.6  **AD** (n = 20)  Age (years) = 79.3; ±5.6  Education (years) = 16.7; ±2.7 | Retention (number of items retrieved in the delayed free recalldivided by the number of itens retrieved in the third free recall of the learning trial); IFR | __ | Retention remains intact in the preclinical phases of AD. IFR presents losses in the preclinical phase |
| Pasquier et al., 2001 | France | cross-sectional | To investigate and compare memory patterns and cerebral perfusion in patients with AD and FTD using neuropsychological tests, including the FCSRT-IR and SPECT. | Words | **Total sample (n = 51)**  **Controls (n = 12)**  Age (years): 69.9; ±3.8  Education (years): ≤ 8 = 8; > 8 = 4  **FTD (n = 14)**  Age (years): 68.1; ±3.8  Education (years): ≤ 8 = 4; > 8 = 10  **AD – Parietal variant (n = 15)**  Age (years): 71.2; ±4.7  Education (years): ≤ 8 = 8; > 8 = 7  **AD – Frontal variant (n = 10)**  Age (years): 72.0; ±6.7  Education (years): ≤ 8 = 8; > 8 = 2 | IFR; ITR; DFR; DTR; number of intrusions; Recognition (yes-no) | __ | The AD group recalled fewer words in all FCSRT-IR mesures (immediate and delayed) than the FDT group and controls, which did not differ from each other. This group also had more intrusions and recognized fewer words than controls; The number of false positives was higher in patients with FDT and AD than in controls. |
| Sarazin et al., 2007 | France | Longitudinal  (3 years) | To determine the effectiveness of various cognitive tests (FCSRT-IR, MMSE, Isaac Set Test, Benton Visual Retention Test, DENO 100, Verbal Fluency, Serial Digit Ordering Test, and Baddeley's Double Task, Similarities - Wais III, Stroop Test, TMT A/B, and Digit Symbol - Wais III) in identifying prodromal AD among patients with MCI. | Words | **Total - MCI (n = 217)**  Age (years): 72; ±5,4  Education (years): 10.8; ±4  **MCI – non AD (n = 158)**  Age (years): 70.9; ±5.4  Education : 44.3% - bachelor´s degree  **MCI – AD (n = 59)**  Age (years): 74.8; ±4.1  Education: 39% bachelor´s degree | IFR, ITR ; DFR; DTR, number of intrusions; ISC | **ITR** **(cutoff = 40)**  Se = 79.7% ; Sp = 89.9%  AUC = 0.94  **ISC** **(cutoff = 71)**  Se = 78% ; Sp = 84.8%  AUC= 0.93  **IFR** **(cutoff = 17)**  Se = 71.2% ; Sp = 91.8%  AUC= 0.92  **DFR =** **(cutoff = 6 )**  Se = 76.3%; Sp = 90.5%  AUC= 0.92  **DTR** **(cutoff =**  **14)**  Se = 69.5% ; Sp = 88.6%  AUC= 0.89  **Intrusions (cutoff = 2)**  Se = 64.4% ; Sp = 85.4%  AUC= 0.87 | All scores from the FCSRT-IR showed the highest accuracy to detect AD compared to the other tools. Among the FCSRT-IR variables, the ITR showed higher sensitivity and the IFR higher specificity. |
| Grober et al., 2008 | USA | Cross-sectional | Evaluate a two-phase strategy for early dementia detection, including an initial screening phase and a subsequent confirmation phase with FCSRT – IR. | Words | **Total sample (n = 318)**  **No-dementia (n = 262)**  Age (years): 78.2; ±7.0  Education (years): 12.9; ±3.2  **Dementia (n = 56)**  Age (years): 81.3; ±7.2  Education (years): 11.1; ±4.4 | IFR | **IFR (cutoff = 25)**  Se= 86% ; Sp= 73% | The two-stage approach, starting with an initial screening followed by the application of the FCSRT-IR, demonstrated improved accuracy in dementia identification, enhancing the detection of early cases and differentiating between AD and non-AD dementias. |
| Auriacombe et al., 2010 | France | Longitudinal  (5 years) | Assess the validity of the FCSRT-IR in predicting the risk of cognitive impairment and dementia attributable to vascular factors. | Words | **Without dementia (n= 1,464)**  Age (years): > 65  **Dementia at 2 years (n = 23)**  Age (years): 78.6; ±5.5  Education: 47.8% GCE  **Dementia at 5 years (n = 60)**  Age (years): 78.8; ± 3.8  Education: 36.7% GCE | IFR; ITR; DFR; DTR | **Dementia at 2 years**  **IFR <16**  General Dementia  Se = 56.5%; Sp = 89.%  PPV = 8.5; NPV = 99.1  DA  Se = 73.3%; Sp = 88.9%  PPV = 7.2; NPV = 99.7  **ITR**  General Dementia **≤ 34**  Se = 47.8%; Sp = 95.1%  PPV = 15.1; NPV = 99.0  DA **≤32**  Se = 60%; Sp = 96.5%  PPV = 16.7; NPV = 99.5  **Dementia at 5 years**  **IFR. ≤22**  General Dementia  Se = 80%; Sp = 64.3%  PPV = 10.9; NPV = 98.3  AD  Se = 92.3%; Sp = 63.9%  PPV = 8.2; NPV = 99.6  **ITR ≤ 42**  Dementia in General  Se = 56.7%; Sp = 76.9%  PPV = 11.8; NPV = 97  AD  Se = 69.2%; Sp = 76.7%  PPV = 9.4; NPV = 98.6 | IFR e ITR of the FCSRT-IR showed reasonable sensitivity and specificity for predicting AD. Although the PPV was low, the NPV was high. |
| Grober et al., 2010 | USA | Longitudinal  (2,6 years) | Identify prevalent dementia, incident dementia, and distinguish AD dementia from other dementia syndromes through FCSRT measures. | Pictures | **Total sample (n = 244)**  **Dementia-free (n = 194)**  Age (years): 78.3; ±6.9  Education (years): 12.5; ±3.3  **Mild dementia (n = 50)**  Age (years): 82.4; ±6.8  Education (years): 10.6; ±4.3 | IFR; ITR; ISC | **Prevalent Dementia (n= 50)**  **IFR ≤ 24**  Se= 78%; Sp= 90%  **ITR ≤ 46**  Se= 54%; Sp= 90%  **Incident Dementia (n=28)**  Impairments in IFR and ITR indicated 4 times higher odds of developing dementia when compared to those with intact performance on these measures. | IFR was the most sensitive measure to identify prevalent and incident dementia; ITR was the best measure differente AD from other types of dementia. Older participants had an increased risk, while other variables did not interfere. |
| Derby et al., 2013 | USA | Longitudinal  (4 years) | Verify the predictive capacity, sensitivity, specificity, and cutoff scores of IFR from FCSRT-IR and the immediate recall - IR from LM for AD, and also to assess whether APOE e4 status, age, and educational level affected test scores. | Pictures | **Memory complaints without dementia (n= 854)**  Age (years)= 78.8; ±5.4 | IFR; ITR | **FCSRT-IR - IFR**  2 years follow-up: cutoff= 32  Se= 94.1%; Sp= 47.0%  3 years follow-up: cutoff= 29  Se= 87.6%; Sp= 70.0%  4 years follow-up: cutoff=27  Se= 80.9%; Sp= 81.7%  **ML – IR** 2 years follow-up: cutoff= 22  Se= 94.4%; Sp= 39.9%  3 years follow-up: cutoff= 22  Se= 92.9%; Sp= 40.7%  4 years follow-up: cutoff= 22  Se= 88.2%; Sp= 41.1% | The FCSRT-IR demonstrated greater sensitivity and specificity in distinguish those who will develop AD dementia over 2-4 years compared to the LM. When the results of the LM and the APOE e4 gene status were combined with the FCSRT-IR, there was no improvement in the ability to predict the occurrence of incident AD. |
| Kiesmann et al., 2013 | France | Cross-sectional | Determine if the abbreviated approach, which combines the MDS checklist and a more concise neuropsychological assessment (MMSE, Literal Verbal Fluency Test, H&Y Rating Scale, IADL, MDRS, ROCF, FCSRT-IR, and PASAT), is capable of effectively identifying PDD compared to the use of the comprehensive checklist and an extensive evaluation. | Words | **A1 Group - PDD with MMSE<26 and 8-item checklist+ (n = 20)**  Age (years):81.2; ±5.2  Education (years):9.7; ±2.7  **A2 Group - PDD with MMSE ≥ 26 and 8-item checklist<8 (n = 11)**  Age (years): 81.1; ±5.4  Education (years): 9.8; ±3.2  **B Group (n = 9)**  Age: 78.1; ±2.9  Education (years): 12.0; ±4.9 | IR; IFR; ITR | **FCSRT-IR – IFR ≤ 22**  Se= 93.5%  Sp= 77.8%  AUC= 0.855  PPV= 92.9%  NPV= 77.8% | MDRS and IFR from FCSRT-IR was the measure that most differentiated the groups with and without dementia in PD. |
| Lemos et al., 2014 | Portugal | Cross-sectional | Assess the usefulness of the FCSRT-IR in distinguishing patients with bvFTD and those with AD and characterize the memory profile for each condition. | Words | **Cognitively healthy (n= 32)**  Age (years): 68.59; ±1.27  Education (years): 7.06; ±0.86  **bvFDT (n= 32)**  Age (years): 68.56; ±1.19  Education (years): 6.97; ±0.84  **AD (n= 32)**  Age (years): 69.72; ±1.27  Education (years): 6.91; ±0.87 | IFR; ICR; ITR; DFR; DCR; DTR; % Retention ^+^ | **model ITR + DTR:**  accuracy = 78.1%  Se = 90.9% ; Sp = 71.4%, | FCSRT-IR showed good internal consistency total sample and individual clinical groups. bvFTD and AD groups showed significant deficits compared to controls in IFR, ITR; DFR and DTR, but only the AD group showed impairment in ICR and DCR and retention. The FCSRT-IR allowed to identify 95% of patients with AD and rule out 63% of patients with bvFTD. Patients with AD were 25 times more likely to have impaired FCSRT-IR performance than patients with bvFTD. There was also a significant difference between bv-FTD and AD patients on all selected FCSRT-IR measures with worse scores for AD. |
| Mura et al., 2014 | France | Longitudinal  (3 years) | Investigate the sensitivity of various neuropsychological tests (FCSRT-IR, BVRT, DENO 100, Phonemic and Semantic Verbal Fluency, Double Task of Baddeley, Similarities WAIS, TMT A/B, Digit Span WAIS) for prodromal AD. | Words | **MCI** (n = 212)  Age (years): 71,8; ±5,3  Education (Years): 57% = 8, 19% = 9-12, 24% = +12 | IFR; ITR; DTR. | __ | FR (immediate and delayed) and verbal fluency demonstrated greater ability to discriminate cognitive changes in patients with prodromal AD. |
| Bertoux et al., 2015 | France | Cross-sectional | Examine the effectiveness of the FCSRT-IR and the Mini-SEA in distinguishing patients with bvFTD and AD. | Words | **Total sample (n = 96)**  **Controls (n = 30)**  Age (years): 67.2; ±8.7  Educational (years): 10.7; ±3.7  **AD (n = 28)**  Age (years): 70.3; ±11.1  Education (years): 11; ±3.6  **A-bvFTD (n = 38)**  Age (years): 66.6; ±9.3  Education (years): 10.9; ±3.8 | IR, IFR; ITR; DTR | **FCSRT-IR (bvFTD x DA):**  AUC: 0.773 | FCSRT-IR was not able to distinguish bvFTD from AD, classifying only 69.7% of patients correctly. Although bvFTD performed better than AD, there was a 53% overlap, making precise differentiation impossible. |
| Grober et al., 2015 | USA | Longitudinal  (5 years) | investigate if the FCSRT-IR outperforms the LM in early dementia detection over five years. | Pictures | **Total sample (n = 836)**  Age (years): 76.8; ± 4.7 (70 a 89)  Education (years): 14.3; ± 3.1 (3 a 13)  **Grups:**  **Conventional (n = 418)**  Age (years): 78.2; ± 4.7  Education (years): 14.1; ± 3.2  **Robust (n = 340)**  Age (years): 77.4; ± 4.5  Education (years): 14.4; ± 3.0  **Incident Dementia (n = 78)**  Age (years): 81.6; ± 4.4  Education (years): 13.7; ± 2.8 | IFR (FCSRT-IR) and IR (LM) | __ | The norms derived from the robust normative sample were higher than those derived from the conventional normative sample, resulting in greater sensitivity to dementia. |
| Lemos et al., 2015 | Portugal | Cross-sectional | Characterize memory dysfunctions in patients with MCI and AD with the FCSRT-IR. | Words | **Total sample (n = 271)**  **Controls (n =101 )**  Age (years):70.22; ±0.76  Education (years): Median = 4 (4 - 11)  **MCI (n = 100)**  Age (years): 71.08; ±0.83  Education (years): Median = 4 (4 - 9)  **AD (n=70 )**  Age (years): 72.63; ±0.98  Education (years): Median = 4 (4 - 11) | IFR; ICR; ITR; DFR; DCR; DTR | **MCI:**  ITR ≤ 35  Se= 72%; Sp= 83%  AUC= 0.818  PPV= 81%; NPV= 75%  DTR ≤ 12  Se= 76%; Sp= 81%  AUC= 0.828  PPV= 80%; NPV= 77%  **AD:**  ITR ≤ 27  Se= 94%; Sp= 99%  AUC= 0.987  PPV= 99%; NPV= 95%  DTR ≤ 8  Se= 96%; Sp= 97%  AUC= 0.991  PPV= 97%; NPV= 96%  **MCI x AD:**  ITR ≤ 21  Se= 84%; Sp= 71%  AUC= 0.844  PPV= 74%; NPV= 82% | The FCSRT-IR demonstrated good diagnostic accuracy in differentiating MCI and AD. TR (immediate and delayed) was the most effective measure to distinguish MCI and AD from controls. There was no significant effect of educational level on the FCSRT-IR results. |
| Lemos et al., 2015 | Portugal | Cross-sectional | To analyze the performance of patients with MCI and AD on the LM, VPAL, and FCSRT-IR and determine which test shows the highest accuracy in classifying MCI and AD. | Words | **Total** **sample (n = 128)**  **aMCI (n = 85)**  Age (years): 70,34; ±0,84  Education (years): 6,81; ±0,49  **AD (n = 43)**  Age (years): 72,07; ±1,23  Education (years): 8,07; ±0,75 | IFR; ITR; DTR. | **LM**  **DFR - cutoff ≤ 2.5**  Se = 91%; Sp = 69%  PPV = 75%; NPV = 88%  **FCSRT-IR**  **IFR – cutoff ≤ 21**  Se = 77%; Sp = 74%  PPV = 75%; NPV = 76%  **DTR – cutoff ≤ 8**  Se = 93%; Sp = 57%  PPV = 68%; NPV = 89% | FCSRT-IR demonstrated superior performance in classifying patients with MCI and DA, regardless of the level of education. |
| Delgado et al., 2015 | Chile | Cross-sectional | Compare the psychometric properties and the ability of the memory measures of the verbal and visual versions of the FCSRT-IR to distinguish patients with mild AD from cognitively healthy controls. | Words/ Pictures | **Cognitively Healthy Controls (CDR = 0)**  Age (years): 71; ± 6  Education (years): 13; ± 4  **Patients with Mild Alzheimer's Disease (CDR = 0.5 and CDR = 1)**  Age (years): 74; ± 6  Education (years): 12; ± 5  **CDR = 0.5**  Age (years): 73 ± 5  Education (years): 11 ± 5  **CDR = 1**  Age (years): 74 ± 7  Education (years): 12 ± 5 | IFR; ITR. | **Words**  **IFR ≤ 22**  Se= 98%; Sp= 91%  AUC= 0.980  **ITR ≤ 39**  Se= 88%; Sp= 95%  AUC= 0.972  **Pictures**  **IFR ≤ 26**  Se= 92%; Sp= 93%  AUC= 0.960  **ITR ≤ 46**  Se= 88%; Sp= 91%  AUC= 0.930 | Although there were score differences between the word and picture versions of the FCSRT-IR within the same group of patients with mild AD and controls, both versions demonstrated good to excellent diagnostic utility, with sensitivities above 90% for IFR) and above 85% for ITR and specificities above90% for both versions. |
| Cerciello et al., 2017 | Italy | Cross-sectional | Determine if the FCSRT-IR provides information for the differential diagnosis between dementia in AD, FTD, and VaD.". | Pictures  (24 items) | **Total sample:**  Age (years): 74,33; ± 5,75  Education (years): 8,58; ± 3,74  **Controls ( n = 20)**  Age (years): 73,3; ± 5,8  Education (years): 9,4; ± 3,8  **DA (n = 15)**  Age (years): 75,1 years; ± 5,3  Education (years): 7,5; ± 3,7  **VaD (n = 10)**  Age (Years): 74,6; ± 5,9  Education (years): 6,9; ± 2,6  **FTD (n = 9)**  Age (years): 72,3; ± 6,4  Education (years): 9,5; ± 3,2 | IFR; ITR; DFR; DTR; ISC for all immediate and delayed trials (ISC 1, 2 and 3 and delayed ISC) | __ | Patients with VaD and FTD benefited from cues more than those with AD. AD and Vad demonstrated a higher forgetting rate in immediate trials (ISC 1,2 and 3) compared to patients with FTD and controls. There was no significant difference in forgetting rate between groups in delayed trial compared to immediate trials. Patients with VaD showed lower ISC in delayed trial compared to immediate attempts, revealing accelerated forgetting in this group. |
| Lemos et al., 2017 | Portugal | Longitudinal  (2 years) | Determine which instrument, including the FCSRT-IR, presents the highest predictive value for the progression of patients with MCI to AD. | Words | **Total sample** **(n = 88)**  **aMCI - aMCI (n = 59)**  Age (Years): 69.63; ±1.05  Education (years): 6,29; ±0,54  **aMCI - AD (n = 29)**  Age (years): 67; ±3.76  Education (years): 7.03; ±0,83 | ITR; DTR. | **ITR ≤ 27 and DTR ≤ 8**  AD conversion rate of 57% | The only variable that remained significantly associated with the risk of conversion to dementia during follow-up was the ITR (FCSRT-IR). The FCSRT-IR showed better predictive validity compared to WMS LM IR, for detecting prodromal AD. |
| Grande et al., 2018 | Italy | Longitudinal  (2 years) | Evaluate the diagnostic accuracy of the FCSRT-IR in predicting the development of AD in individuals with MCI. | Pictures  (12 items) | **Total sample (n = 187)**  Age (years): 75.5; ±6.8  Education (years): 7.6; ±3.7  **Non-converters**  **at follow-up (n= 100)**  Age (years): 74.3; ±7.3  Education (years) 7.4; ±3.6  **AD at follow-up (n = 73)**  Age (years): 76.4; ±6.1  Education (years): 8.0; ±3.7  **Non-AD dementia**  **at follow-up (n = 14)**  Age (years): 78.8; ±4.6  Education (years): 7.6; ±3.9 | IFR; ITR; DFR; DTR; ISC | **IFR**  AUC= 0.71  Se= 75.3%; Sp= 66.7%  PPV= 80.9%; NPV= 59.1%  **ITR <35**  AUC= 0.72  Se= 71.2%; Sp= 61.9%  PPV= 85.3%; NPV= 79.6%  **DFR**  AUC= 0.76  Se= 80.8%; Sp= 71.1%  PPV= 85.3%; NPV= 64.1%  **DTR <11**  AUC= 0.70  Se= 56.2%; Sp= 83.3%  PPV= 74.8%; NPV= 68.3%  **ISC < 0.9**  AUC= 0.69  Se= 65.8%; Sp= 71.9%  PPV= 76.6%; VPN= 60% | The FCSRT-IR proved to be a highly predictive tool for AD development in individuals with MCI, exhibiting greater specificity when combined with category fluency tests. MCI individuals who scored below FCSRT-IR cutoff scores were diagnosed with AD 2-3 years earlier than those who tested negative, highlighting its utility in the early identification and monitoring of these patients. |
| Perri et al., 2019 | Italy | Cross-sectional | Evaluate how the FCRST differentiates between SIVD and AD by analyzing memory mechanisms and the impact of support on encoding and retrieval. | Pictures | **Total sample (n = 57)**  **Controls (n = 20)**  Age: 76.5; ±1.2  Education (years): 10.0; ±1.0  **AD (n = 20)**  Age (years): 76.4; ±1.1  Education (years): 8.9; ±1.1  **SIVD (n = 17)**  Age: 77.9; ±1.3  Education (years): 10.0; ±1.1 | IFR; ITR; DFR; DTR. | __ | SIVD showed better performance in the FCSRT-IR encoding and retrieval of information when compared to the AD group. SIVD had better performance in DTR and DFR than AD group. When associated with the 15-word list test there was an improvement in diagnostic accuracy. |
| Zibetti et al., 2019 | Brazil | Cross-sectional | Present the process of adaptation of the FCSRT-IR to Brazilian population and evidence of clinical, criterion and concurrent validity | Pictures | **Total sample (n = 50)**  Age (years):: 73.26; ±6.7  Education (years): 10.8; ±5.76  **Controls (n = 25)**  Age (years): 72.68; ±6.54  Education (years): 12.44; ±5.63  **AD (n = 25)**  Age (years): 73.84; ±6.94  Education (years): 9.16; ±5.3 | ICR, FR (1, 2, 3 trials); TR (1, 2, 3 trials); DFR; DTR; TFR, TR | **DA**  ICR – cutoff ≤15  Se= 64%,; Sp= 100%  AUC= 0.820  Trial 1 IFR – cutoff ≤6  Se= 84%; Sp= 84%  AUC=0.928  Trial 1 ITR - ≤15  Se= 88%; Sp= 96%  AUC= 0.934  Trial 2 IFR ≤6  Se= 92%; Sp= 100%  AUC= 0.978  Trial 2 ITR - ≤15  Se= 68%; Sp= 100%  AUC= 0.840  Trial 3 IFR - ≤5  Se= 80%; Sp= 100%  AUC: 0.958  Trial 3 ITR - ≤15  Se= 72%; Sp= 96%  AUC= 0.853  DFR - ≤ 8  Se= 92%; Sp: 97%  AUC: 0.991  DTR - ≤15  Se= 72%; Sp= 96%  AUC= 0.867  IFR - ≤21  Se= 92%; Sp= 96%  AUC= 970  ITR - ≤47  Se= 92%; Sp= 96%  AUC= 0.950 | FCSRT-IR demonstrated high sensitivity and specificity in identifying patients with AD. Evidence of concurrent and criterion validity, demonstrated it is a valuable tool for assessing memory in clinical populations. |
| Garcia-Gutierrez et al., 2022 | Spain | Cross-sectional | Develop machine learning-based models to diagnose AD and bvFTD, as well as for the differential diagnosis between them, using a lot of cognitive tests (ACE-III; Digit span; FCSRT-IR; ROCF; Corsi Cubes; TMT A/B; SDMT; SCWIT, ToL; BNT; Semantic and Letter fluency; JLO and VOSP). | Words | **AD (n= 170)**  Age (years): 73.39; ±8.13  Education (years): 9.55; ±4.83  **bvFTD (n= 72)**  Age (years): 71.33; ±7.5  Education (years): 9.22; ±4.29  **HC (n= 87)**  Age (years): 70.69; ±8.59  Education (years): 11.23; ±4.6 | IFR (1^st^ trial); IFR; TFR; DFR; DTR | **__** | FCSRT- IR was identified as one of the most important predictors to distinguish between AD, bvFTD and healthy controls,. A IFR score ≤ 7 was identified as a possible indicator of AD or bvFTD, depending on the results of ROCF, VF and education. |
| Grober et al., 2021b | USA | Longitudinal  (20 years) | Verify the predictive validity of SOMI stages (0-4) in relation to AD neuropathology. | Pictures | **Subjects + and - for pathology AD (n=251)**  **neuropatology + AD (n = 159)**  Age (years): 87.9; ±10.1;  Education (years): 15.2; ±2.9  **neuropatology – AD (n = 92)**  Age (years): 89,4; ±9,0  Education (years): 14.6; ±3.1 | SOMI score | SOMI score  AUC= 0.95 | The SOMI test proved to be a strong predictor for Alzheimer's neuropathology. Participants with moderate (SOMI 4) or severe (SOMI 5) impairment had significantly higher chances of presenting positive AD neuropathology. SOMI outperformed the CDR-SB in prediction accuracy. |
| Grober et al., 2022 | USA | Longitudinal  (10 years) | Compare the predictive value of IFR and DFR from the FCSRT-IR for MCI. | Pictures | **Total sample (n = 1283)**  Age (years): 69.57; ±8.29  Education (Years): 16.69; ±2.66 | IFR; DFR | **IFR:**  Model 1: HR = 0.662  Model 3: HR = 0.767  **DFR:**  Model 2: HR = 0.676  Model 3: HR = 0.808 | IFR and DFR are independent and significant predictors of MCI risk, suggesting that both learning and retention abilities are important for early identification of this condition. The addition of DFR to the IFR model provides a marginal improvement in MCI prediction, which may justify its inclusion depending on the clinical context and time constraints. Age was a significant predictor of MCI, while education and sex were not. |
| Montesinos et al., 2022 | Peru | Cross-sectional | Validate the psychometric properties of the picture version of the FCSRT-IR among illiterates in the Callao region, Peru. Compare the performance of IFR and ITR of the FCSRT-IR among three groups: cognitively healthy individuals, MCI, and patients with early-stage AD, compared to performance on the RUDAS-PE. | Pictures | **Total sample (n = 187)**  Age (years): 70,2; ±3,8  **Cognitively Healthy (n = 64)**  **aMCI (n = 60)**  **AD (n = 63)** | IFR; ITR | **IFR**  Controls X aMCI: cutoff = 16  Controle X early AD: cutoff = 10  CCLa X early DA: cutoff = 10  **ITR**  Controls X aMCI: cutoff = 26  Controle X early AD: cutoff = 20  CCLa X early DA: cutoff = 19  Controle X CCLa: Nota de corte = 26  * "Youden index = 1.00 for all measures." | The FCSRT-IR was significantly better at discriminating normal controls from MCI when compared to the RUDAS-PE in the illiterate population of Peru. FCSRT-IR measures were not sensitive enough to discriminate MCI from dementia in AD in this population. There was no significant influence of sex and years of education. |
| Mura et al., 2022 | France | Longitudinal  (5 years) | Investigate the ability of various cognitive tests (MMSE, TMT, FCSRT-IR, verbal fluency, CDR-SB) to predict the onset of dementia within a 3 to 5-year follow-up period in patients with memory complaints without dementia. | Words | **Total sample (n = 860)**  Age (years): 75.8; ±4.2)  Education: No diploma - 19.7%;  Primary school certificate - 44.0%; Secondary education, without high school diploma - 17.8%, High school diploma (Baccalaureate) or higher - 18.6%  APOE genotype: Ɛ4 carrier 25.2%  **Dementia (n = 49)**  Age (years): 77.2; 3.7  Education: : No diploma – 24.5%;  Primary school certificate – 46.9%; Secondary education, without high school diploma – 12.2%, High school diploma (Baccalaureate) or higher – 16.3%  APOE genotype: Ɛ4 carrier 35%  **without Dementia (n = 811)**  Age (years): 75.7; ±4.2  Education: No diploma – 19.4%;  Primary school certificate – 43.7%; Secondary education, without high school diploma – 18.1%, High school diploma (Baccalaureate) or higher – 18.7%  APOE genotype: Ɛ4 carrier 24.6% | IFR; ITR; DFR; DTR. | **Change in IFR (Y2–Y0) in dementia prediction:**  AUC= 0.72  Se= 65%; Sp= 71%  PPV= 13%; NPV= 97%  **IFR at the 2-year visit (Y2) in dementia prediction**   - AUC= 0.89   Se= 89%; Sp= 83%  PPV= 20%; NPV= 99% | IFR between year 0 and year 2, and at the 2-year visit were significant predictors of AD, even after adjustments for age, sex, level of education, CDR at baseline, and APOE ε4 allele. |
| Bello-Lepe et al., 2023 | Chile | Cross-sectional | Verify the diagnostic accuracy of the FCSRT -IR to identify MCI and dementia. | Pictures | **HOA (n = 113)**  Age (years): 71.9; ±7.64  Education: 46,9% com entre 1 e 12 years de educação formal, 53,1% com mais de 12 years de educação formal  **MCI (n = 65)**  Age (years): 76.9 ±8.71  EscolarAge: 67,7% com entre 1 e 12 years de educação formal, 32,3% com mais de 12 years de educação formal  **Dementia (n=48)**  Age (years): 82.17; ±7.89  EscolarAge: 83,3% com entre 1 e 12 years de educação formal, 16,7% com mais de 12 years de educação formal | IFR; ITR; DFR; DTR; ISC | **MCI vs. HOA:**  Se= between 0,342 and 0,458  Sp= between 0,912 and 0,947  AUC= between 0.645 and 0.702  **Dementia vs. HOA:**  Se= between 0,837 e 0,899  Sp= between 0,92 e 0,956  AUC= between 0.885 and 0.928  **Dementia and MCI vs. HOA:**  Se= 55.4 - 63.4%  Sp= 88.5 - 91.2%  AUC= 71.9 - 76.8%  **MCI vs. Dementia:**  Se= 58.1 - 73.9%  Sp= 84.4 - 96.9%  AUC= 66 - 77.7% | FCSRT-IR measures obtained AUCs greater than 0.90 to identify dementia compared to healthy controls. The MoCA and MMSE were more accurate to identify MCI. DTR showed to be the best FCSRT-IR measure. |
| Kuo et al., 2023 | Taiwan | cross-sectional | To obtain the cognitive profiles of eight native Chinese-speaking individuals, suspected of having MCI, AD, bvFTD, or nfaPPA, through a series of neuropsychological tests (ToMT, FCSRT-IR, and others). | Words | **Total sample (n= 9)**  **Control** (n = 1)  Age (years): 64  Education (years): 12  **MCI/AD** (n = 3)  Age (years) = 65; 71; 66  Education (years) = 16; 6; 9  **bvFTD** (n = 2)  Age (years) = 66; 54  Education (years) = 9; 16  **PPA** (n = 3)  Age (years) = 62; 62; 67  Education (years) = 12; 16; 16 | IFR; ITR; DFR; DTR; ISC and delayed ISC (DTR-DFR) | __ | Participants with FTLD (bvFTD and nfvPPA) had lower FCSRT-IR scores relative to participants with MCI/AD. Results from other neuropsychological tests have been inconsistent, suggesting that the FCSRT-IR may be a more sensitive indicator of cognitive difficulties, especially in FTLD conditions. |

Abbreviations: ACE- III, Addenbrooke’s Cognitive Examination; AD, Alzheimer’s Disease; aMCI, amnestic Mild Cognitive Impairment; AUC, Area Under the Curve; bvFTD, Behavioral Variant Fronto Temporal Dementia; BNT, Boston Naming Test; BVRT, Benton Visual Retention Test; DCR, Delayed Cued Recall; DFR, Delayed Free Recall; DTR, Delayed Total Recall; FTD, Fronto Temporal Dementia; GCE, General Certificate of Education; HC, Healthy Control; HOA, Healthy Older Adults; HR, Hazard Ratio; ICR, Immediate Cued Recall; H&Y Rating Scale, Hoehn and Yahr Rating Scale; IADL, Instrumental Activities Dayli Living; IFR, Immediate Free Recall (sum of free recall over the three test trials); ISC, Index of Sensitivity of Cueing; JLO, Judment of Line; LM, Logical Memory from Weschsler Memory Scale; MCI, Mild Cognitive Impairment; MDRS, Mattis Dementia Rating Scale; MDS, Movement Disorder Society; MMSE, Mini Mental State Examination; MoCA, Montreal Cognitive Assessment; naMCI, non-amnestic Mild Cognitive Impairment; NCD= Neurocognitive disorder; nfaPPA, non-fluent agrammatic variant Primary Progressive Aphasia; NPV, Negative Predictive Value; PASAT, Paced Auditory Serial Addition Test; PPA, primary progressive aphasia; FTLD, frontotemporal lobal degeneration; PDD, Parkinson's Disease Dementia; PPV, Predictive Positive Value; ROCF, Rey-Osterreith Complex Figure; RUDAS-PE, Rapid Universal Dementia Assessment Scale for Primary Edition; SCWIT, Stroop Colour-Word Interference Test; Se, Sensitivity; SIVD, Subcortical Vascular Dementia; SMDT, Symbol Digit Modalities Test; Sp, Specificity; TMT A/B, Trail Making Test A/B; ToL, Tower of London-Drexel version; VaD, Vascular Dementia; VOSP, Visual object and Space Perception Battery; VPAL, Paired Associative Learning; WAIS, Wechsler Adult Intelligence Scale.

Notes: *ISC= (total recall-free recall)/(48-free recall); + % Retention = [(DTR/3rd ITR) *100].

**Table S3.** Studies which have correlated FCSRT-IR performance with biomarkers for dementia.

| **Author, years** | **Country** | **Study Design**  **(Follow-up years)** | **Objective** | **Version** | **Type of Biomarker Test** | **Sample Characteristics**  **(Mean; SD)** | **Variables**  **FCSRT** | **Results** |
| --- | --- | --- | --- | --- | --- | --- | --- | --- |
| Lavenu et al., 1998 | France | Cross-sectional | To investigate the relationship between performance on the FCSRT-IR and MTL atrophy in patients with FTD. | Word | CT scan | **FTD without MTL atrophy (n = 17)**  Age (years): 66.17; ±7.6  Education (years): <8 y= 12 ; 18 y = 5  **FTD with MTL atrophy (n = 5)**  Age (years): 67; ±6.2  Education (years): <8 y = 2; 18 y = 3 | IFR; ITR; TCR; Intrusions^¶^; Recognition^²^. | MTL atrophy correlated with ITR. Patients with MTL atrophy scored lower on some memory measures, such as ITR and IFR, compared to those without MTL atrophy. The % of TCR was lower, and the number of intrusions was higher in the MTL atrophy group. |
| Toledo-Morrel et al., 2000^1^ | USA | Cross-sectional | To investigate the relationship between hippocampal atrophy and memory dysfunction in patients with AD according to the type of material memorized (verbal or spatial) from FCSRT-IR. | Picture | sMRI | **Total sample**  Age (years): 68.6; (49–84)  **Controls (n= 30 )**  Vol right hippocampus: 1.580, ±0.234  Vol left hippocampus : 1.552, ±0.187  Vol right parahippocampus gyrus: 1.764; ±0.309  Vol left parahippocampus gyrus: 1.901; ±0.322  Vol right temporal lobe: 18.353; ±1.880  Vol left temporal lobe: 18.892; ±2.065  **AD (n= 18)**  Vol right hippocampus: 1.298, ±0.339  Vol left hippocampus: 1.294; ±0.284  Vol right parahippocampal gyrus: 1.689; ±0.294  Vol left parahippocampal gyrus: 1.737; ±0.359  Vol right temporal lobe: 18.891; ±2.520  Vol left temporal lobe: 18.977; ±2.801 | %IFR (3rd trail); %DFR (after 60’); % immediate spatial recall; % delayed spatial recall from FCSRT-IR | Hippocampal atrophy was strongly associated with impairment in IFR and DFR in AD patients. Memory for verbal information of the FCSRT-IR was associated with left hippocampal volume, and spatial memory with right hippocampal volume. No significant relationships were found between parahippocampal gyrus or temporal lobe volume and memory, suggesting a specific association for the hippocampus. |
| Pasquier et al., 2001 | France | Cross-sectional | To investigate and compare memory patterns and cerebral perfusion in patients with AD and FTD using neuropsychological tests, including the FCSRT-IR. | Word | SPECT | **Total sample (n = 51)**  **Controls (n = 12)**  Age (years): 69.9; ±3.8  Education (years): ≤ 8 = 5; > 8 = 4  **FTD (n = 14)**  Age (years): 68.1; ±3.8  Education (years): ≤ 8 = 4; > 8 = 10  **AD – Parietal variant (n = 15)**  Age (years): 71.2; ±4.7  Education (years): ≤ 8 = 8; > 8 = 7  **AD – Frontal variant (n = 10)**  Age (years): 72.0; ±6.7  Education (years): ≤ 8 = 8; > 8 = 2 | IFR; ITR; DFR; DTR; number of intrusions^¶^; Recognition^²^. | The results indicated that while both FTD and AD patients exhibited short-term memory deficits, the nature of these deficits differed. FTD patients benefited more from cues than AD patients, suggesting differences in retrieval strategies, possibly linked to the frontal hypoperfusion observed in SPECT imaging for FTD. |
| Lekeu et al., 2003^2^ | Belgium | Cross-sectional | To investigate how performance on the French version of the FCSRT-IR correlates with resting-state cerebral metabolism patterns in patients with AD. | Word | 18FDG-PET | **Total sample (n=31)**  **Healthy Controls – group 1 (just memory test) (n= 31)**  Age (years): 69.4; ±4.2  **Healthy Controls – group 2 (just PET scan) (n = 20)**  Age (years): 64.45; ±5.79  **AD (n= 31)**  Age (years): 70.9; ±6.1 | IFR (1^st^ trial); ICR (1^st^ trial); DFR; DCR. | The AD group obtained lower scores in all mesures (IFR, DFR, ICR and DCR) and benefited less from cues than healthy controls. Brain activity during IFR in AD patients showed a positive correlation with metabolism in the right superior and the right inferior frontal gyrus, reflecting a strategic retrieval attempt. ICR correlated with residual activity in bilateral hippocampal regions. AD group revealed decreased functional connectivity between parahippocampal and frontal areas, which may contribute to impaired performance in free and cued recalls (IFR, DFR, ICR and DCR). |
| Farlow et al., 2004 | USA | Cross-sectional | To examine the impact of APOE genotypes on cognitive performance in individuals with MCI, correlate hippocampal volume between carriers and non-carriers of the APOE ε4 allele, and use baseline data to understand the probability of conversion from MCI to dementia. | Word | sMRI and APOE genotyping through blood sample | **Total sample (n = 494)**  **Non-carriers ε 4 (n = 296)**  Age (years): 70.8; ±8.1  Education (years): 11.2; ±4.3  **One ε4 allele (n = 154)**  Age (years): 70.8; ±6.8  Education (years): 11.1; ±4.0  **Two ε4 allele (n = 44)**  Age (years): 69.8; ±6.7  Education (years): 11.3; ±4.2 | ITR | Participants carrying the APOE ε4 allele (both with one and two alleles) showed lower hippocampal volume compared to non-carriers of the ε4 allele. Carriers performed worse on the ITR of the FCSRT-IR compared to non-carriers of ε4 allele. |
| Zimmermann et al., 2008 | USA | Cross-sectional | To evaluate hippocampal volumes through MRI and hippocampal metabolic measures (NAA/Cr) as predictors of performance on the FCSRT-IR in healthy older adults. | Word | sMRI and MRS | **Total sample (n = 48)**  Age (years): 81.18; ±5.47  Education (years): 13.08; ±3.11  **Controls (n = 34)**  Age (years): 80.37; ±5.77  Education (years): 13.2; ±3.5  **aMCI (n = 14)**  Age (years):83.14;±4.2  Education (years): 12.7; ±1.9 | IFR; ITR | Poorer performance on the IFR of the FCSRT-IR was associated with smaller hippocampal volumes and lower NAA/Cr levels. The same results were not observed with the Logical Memory test . |
| Godefroy et al., 2009 | France | Cross-sectional | To determine patterns of memory impairment associated with specific brain lesion locations in post-stroke patients. | Word | sMRI e CT scan | **Total sample (n = 73)**  Age (years): 65 (22 – 88)  Education (years): < 9 y = 35; 9-11 y = 23; > 11 y = 15 | IR ^**^; ;IFR; ITR; DFR; DTR; Learning curve^+ +^; Intrusions^¶^; Recognition^²^, Recall consistency on free recall^¹^, Forgetting rate **^§^** (3^rd^ trial)*; False Recognition^+^, Repetitions of the same item | Lesions in the medial temporal lobe, thalamus, frontal lobes, centrum semiovale, and striatum were correlated with memory deficits in various measures of the FCSRT-IR, especially in IFR, ITR, DFR and DTR, predominantly in the left hemisphere. Inefficient cueing or false recognitions were associated with frontal damage, highlighting the crucial role of the prefrontal cortex in strategic mnemonic processes. |
| Sanchez-Benavides et al., 2010 | Spain | Cross-sectional | To verify the correlation between performance on neuropsychological tests, including the FCSRT-IR, with hippocampal and entorhinal cortex volumes in healthy controls, MCI and AD. | Word | sMRI | **Controls (n = 34)**  Age (years): 71.7; ±5.9  Education (years): 10.6; ±6.0  **MCI (n = 24)**  Age (years): 74.3; ±6.2  Education (years):8.12; ±4.6  **AD (n = 20)**  Age (years): 75.3; ±6.6  Education (years):7.2; ±4 | ITR; DTR at Half an hour. | DTR and ITR showed significant correlations with hippocampal and entorhinal cortex volumes, especially in participants with MCI. In the AD group, only the DTR showed a moderate correlation with left hippocampal volume. The FCSRT-IR proved effective in discriminating between groups, especially between healthy controls and AD. |
| Habert et al., 2011 | France | Longitudinal  (3 years) | To verify the effectiveness of using SPECT combined or not with FCSRT-IR scores for the early detection of AD. | Word | SPECT | **Total sample** (n=251)  **MCI-S (n =72)**  Age (years): 70.8; ±5.3  Education (years): 12; ±4.2  **MCI-AD (n =11)**  Age (years): 76.2; ±3.8  Education (years): 11.9; ±3.8 | IFR; ITR; DTR. | When combined with IFR and ITR scores, SPECT parameters showed better accuracy in detecting AD. |
| McLaren et al., 2012 | USA | Longitudinal  (6 months) | To identify changes in regional fMRI-BOLD that are related to alterations in neuropsychological performance in patients with mild AD. | Word | fMRI-BOLD | **AD (n= 24)**  Age (years): 71.63; ±1.71  Education (years):16.0; ±0.57 | IFR; ITR | There were positive correlations between changes in IFR and changes in fMRI-BOLD signals in the pre and post-central gyri, inferior parietal lobe, left precuneus, right middle frontal gyrus, right middle cingulate gyrus, paracentral lobule, left posterior cingulate, fusiform gyrus, and left and right hippocampus and parahippocampus. IFR is useful to assess the progression of AD. |
| Wagner et al., 2012 | Germany | Cross-sectional | To assess how cued recall measures relate to a biomarker profile indicative of AD in CSF compared to other memory and non-memory tests in patients with MCI. | Word | CSF  (Aβ42, t-tau; p-tau181) | **Total sample (n = 185)**  **MCI/CFS + (n= 74)**  Age (years): 69.9; ±7.7  Education (years): 12.8; 3.2;    **MCI/CSF - (n=111)**  Age (years): 63.3; ±8.1  Education (years): 12.5; ±3.1 | IFR; ITR; ITR (1^st^ trial); DTR. | ITR correlated more strongly with AD biomarkers compared to Logical Memory and the word list from the CERAD-NP. |
| Perri et al., 2015 | Italy | Cross-sectional | To verify memory profiles in the FCSRT-IR for AD and non-AD in individuals with different kinds of MCI (single and multiple domain, amnestic and non-amnestic) and compare brain morphostructural changes evidenced by magnetic resonance imaging in patients with memory profiles for AD and non-AD from the FCSRT-IR | Picture  (24 items) | sMRI | **Total sample (n = 47)**  **Controls (n = 20)**  Age (years): 73.2; ±4.0  Education (years): 10.2; ±3.7  **aMCIsd (n = 18)**  Age (years): 69.2; ±6.4  Education (years): 12.3; ±4.4  **aMCImd (n = 10)**  Age (years): 69.1; ±6.5  Education (years): 9.1; ±4.8  **naMCI (n = 9)**  Age (years): 72.9; ±6.2  Education (years): 8.4; ± 3.2 | DFR; ISC^\|\|^ (cued recall-free recall)/(total recall-free recall) | Patients with aMCIsd with lesser cueing benefit showed patterns of cerebral atrophy consistent with early stages of AD. The ISC score was useful in differentiating individuals with MCI who were likely to convert to AD. |
| Slachevsky et al., 2017 | Chile | Cross-sectional | To explore the associations between performance on the two versions of the FCSRT-IR and atrophy of cortical structures in patients with mild AD and cognitively normal controls. | Word  Picture | sMRI | **Total sample (n=69)**  **Controls (n = 34 )**  Age (years): 72; ±5.8  Education (years): 12.94; ±3.77  **Mild AD (n = 35 )**  Age (years): 74.23; ±6.59  Education (years): 11.06; ±4.99 | IFR; ITR | There were significant correlations between the Word and Picture versions of the FCSRT-IR and atrophy in different cortical regions. The IFR of the word version were associated with the right middle frontal gyrus. IFR of the picture version were primarily associated with the right temporal fusiform gyrus and parahippocampal region. Performance on the Picture version was higher compared to the Word version in both AD patients and controls. |
| Teichmann et al., 2017 | France | Cross-sectional | To determine the efficacy of the FCSRT-IR in distinguishing AD from SCD and depression, using CSF biomarkers to characterize AD pathology. | Word | CFS  (t-tau; p-tau181; Aβ1-42) | **Total sample (n = 650)**  Age (years): 67.6; ±9  Education (years): 11.1; ±4.5  **AD dementia (n = 200)**  Age (years): 68; ±0.7  Education (years): 10.5; ±0.3  **AD prodromal (n = 16)**  Age (years): 72.5; ±2.3  Education (years): 12; ±1.2  **FDT (n= 69)**  Age (years): 67.1; ±1.1  Education (years): 11.4; ±0.5  **CBS (n= 59)**  Age (years): 69.7; ±1.2  Education (years): 10.5; ±0.6  **PSP (n= 16 )**  Age (years): 70.4; ±2.4  Education (years): 11.9; ±1.2 | IFR; ITR; DFR; DTR; Recognition^²^; False Recognition^+^; Intrusions^¶^; ISC^\|\|^ | Abnormally low FCSRT-IR scores, especially in IFR and ITR scores, demonstrated high sensitivity (100%) to detect AD and prodromal stages, albeit with lower specificity (74.8%), while the number of intrusions proved to be a significantly marker of cognitive impairment, with an effective discriminative threshold of four intrusions to distinguish AD from other groups, with a sensitivity of 83% and specificity of 72%. |
| Arighi et al., 2018 | Italy | Cross-sectional | To evaluate differences between the Word and Picture versions of the FCSRT-IR and their correlation with cortical atrophy in patients with MCI. | Word  (16 items)  Picture  (12 items) | sMRI | **MCI (n=14)**  Age (years): 71.9; ±6.8 | IFR; ITR; ISC^\|\|^. | IFR showed correlation with neuroimaing parameters. The word version correlated with hippocampal atrophy, while the picture version correlated with atrophy in the fusiform gyrus and the visual cortex. |
| Spallazzi et al., 2020 | Italy | Cross-sectional | To investigate the association between FCSRT-IR scores and amyloid load in the precuneus and posterior cingulate cortex in a sample of patients with MCI (single and multiple domain, amnestic and non-amnestic) and mild dementia. | Picture  (12 items) | Amyloid- β PET | **Total sample (n = 79)**  Age (years): 70.4; ±8.1 (42–88)  Education (years): 10.3; ± 4.6  *aMCIsd= 17; naMCIsd= 12; aMCImd= 27; naMCImd= 15; mild dementia= 8  **< 76 years (n = 54)**  Age (years): 67.13; ± 7.1 (42 – 75)  Education (years): 9.67; ± 4.4  ***aMCIsd=** 10; **naMCIsd=** 8; **aMCImd=** 22; **naMCImd**= 10; **mild dementia**= 4 | IFR; ITR; DFR; DTR; ISC^\|\|^; Intrusions^¶^. | The predictive ability of the FCSRT-IR (IFR and DFR) for amyloid pathology appeared to be more effective in younger patients and decreased in patients over 75 years old. ITR also proved to be a good predictor in younger patients, reflecting early hippocampal dysfunction. Lower scores on ITR and ISC correlated negatively with amyloid burden, especially in patients <76 years old. Especially in the group of patients aged **< 76 years**, less than 15% of individuals who showed positive amyloid uptake in the precuneus/posterior cingulate cortex obtained high scores in IFR. The FCSRT-IR was better able to identify patients with AD when dysfunction in the precuneus/posterior cingulate cortex was considered. |
| Vergallo et al., 2020 | France | Cross-sectional and Longitudinal  (3 years) | To assess whether age, sex, and APOE ε4 allele influence plasma YKL-40 concentrations and its association with brain amyloid-β deposition, neuronal activity, neurodegeneration and cognitive performance including FCSRT-IR at baseline and after 3 years of follow-up. | Word | Plasma YKL-40, Amyloid-β  PET and 18FDG-PET,  sMRI. | **Baseline (n = 314)** Age (years): 76.07; ±3.51  Gender (n): 200 females  1-year follow-up = 137 females  3- year follow-up = 89 females | ITR | The FCSRT-IR and YKL-40 concentrations showed a positive association, suggesting that elevated levels may play a protective role against cognitive decline; however, due to the small effect size, this interpretation should be approached with caution. Men exhibited higher YKL-40 concentrations than women, indicating a potential sex-based dimorphism in the inflammatory response. |
| Grober et al., 2021a | USA | Longitudinal with post-mortem analyses | To verify which cognitive test (FCSRT-IR, MMSE, CDR-SB) had the highest correlation with Braak stages based on their last assessment prior to death. | Picture | Histological examination  (Braak stages) | **Total sample (n = 315)**  Age (years): 82.6; ±7.2  Education (years): 16,6; ±2,8x | IFR; ITR | Performance on the IFR declined progressively from Braak stages III and IV, while ITR and CDR-SB showed significant decline only from stage IV. MMSE showed an initial decline at stage IV and a further decline at stage VI. FR and TR were significant predictors of higher Braak stages, surpassing the effectiveness of MMSE and CDR-SB in predicting Braak stages. Impairments in IFR showed a higher correlation with an early burden of pathological tau. |
| Grober et al., 2021b | USA | Longitudinal  (20 years) | To verify the predictive validity of SOMI stages (0-4) in relation to AD neuropathology and the discriminative capacity of Braak stages compared to CDR-SB. | Picture | Histological examination  (Braak stages) | **Subjects + and - for pathology AD (n=251)**  **neuropatology + AD (n = 159)**  Age (years): 87.9; ±10.1;  Education (years): 15.2; ±2.9  **neuropatology – AD (n = 92)**  Age (years): 89,4; ±9,0  Education (years): 14.6; ±3.1 | SOMI | The SOMI test proved to be a strong predictor for Alzheimer's neuropathology and Braak stages. Participants with moderate (SOMI 4) or severe (SOMI 5) impairment had significantly higher chances of presenting higher Braak stages. SOMI outperformed the CDR-SB in prediction accuracy. |
| Perticone et al., 2021 | Italy | Cross-sectional | To investigate whether hyperglycemia one hour after glucose loading (plasma glucose ≥155 mg/dL) negatively affected subcortical regions of the brain, as well as memory tests, in a small cohort of glucose normotolerant individuals. | Colored Picture  (12 items) | MRI | **Total sample (n= 32)**  Age (years): 60.4; ±2.8  **NGT 1-h-high (n- 17)**  Age (years): 59.8; ±3.3  **NGT 1-h-low (n= 15)**  Age (years): 61.3; ±2.5 | IFR; ITR; DFR; DTR. | Individuals with NGT 1-h-high exhibited smaller brain volumes in the cerebellum, hippocampus, and amygdala, higher mean diffusivity in the hippocampus and caudate nucleus, and significantly poorer performance on various memory tests, with pathological results in immediate recall (RAVLT); DFR and DTR (FCSRT). |
| Puttaert et al., 2021 | USA | Cross-sectional | To compare hippocampal volume, glucose metabolism, and spectral parameters of alpha activity in healthy older adults with performance on the FCSRT. | Word | MEG, sMRI, and FDG-PET | **Total sample (n= 37)**  **Controls (n = 19)**  Age (years):64.31; ±7.6 (50 – 90)  Education (years):14.26; ±4.28  **aMCI (n = 19)**  Age (years):71.22; ±5.9  Education (years): 12; ±3.8  **ACS (n = 18)**  Age (years): 74.94; ±8.22  Education (years): 12.77; ±3.52 | IFR; ITR; DFR (20’); DTR; DFR (1 week); DTR (1 week); Encoding Impairment* index*; Long-term retention rate index^…^; ISC^\|\|^. | The peak alpha frequency was positively correlated with ISC. Patients showed lower rates of encoding and long-term retention compared to healthy older adults, suggesting memory dysfunction related to decreased alpha activity and pathological aging |
| Grober et al., 2022 | USA | Cross-sectional | To investigate whether the SOMI stages (0-4) correlate with amyloid load and brain atrophy. | Picture | Amyloid PET and sMRI | **Healthy controls (n = 4484)**  Age (years): 71,3; ±4,6  Education (years): 16,6; ±2,8 | SOMI | Higher stages of SOMI were associated with higher amyloid pathology burden and smaller volume of the hippocampus, entorhinal cortex, and lower temporal lobes. |
| Garo-Pascual et al., 2023 | Spain | Longitudinal  (8 years) | To characterize the brain structure of superagers and identify demographic, lifestyle, and clinical factors, with cognitive assessments including the FCSRT-IR and non-memory tests. | Word | sMRI, blood biomarkers, | **Total sample (n= 37)**  **Superagers (n = 64)**  Age (years): 81.6 (80.4 – 83.1)  Education (years):16.0 (10.0 – 19.0)  **Typical older adults (n = 55)**  Age (years): 82.1 (81.3 – 83.0)  Education (years): 10.0 (6.0 – 17.5) | DFR | Superagers showed significantly superior performance on the DFR compared to typical older adults. Performance on the FCSRT-IR positively correlated with the volume of the hippocampus and medial temporal lobe regions. Superagers exhibited a larger volume in these brain areas and demonstrated a slower rate of gray matter atrophy over time. |
| Giuffrè et al., 2024 | Italy | Cross-sectional | To investigate the differences in FCSRT-IR scores between aMCI with and without evidence of amyloid deposition, predict the A+/A– status based on FCSRT scores, and analyze the relationship between FCSRT-IR performance and biomarkers of amyloidopathy, tauopathy, and neurodegeneration in both groups. | Picture | CSF | **aMCI A+ (n = 90)**  Age (years): 69.37; ±6.06  Education (years): 13.12; ±3.61  MMSE score: 25.20; ±2.66  **aMCI A- (n = 30)**  Age (years): 68.77; ±6.15  Education (years): 13.14; ±4.06  MMSE score: 26; ±2.70 | IFR, ITR, DFR, DTR, ISC^\|\|^. | All FCSRT subitem scores were significantly lower in the A+ group, showed negative correlations with p-tau181 and t-tau levels, and were able to predict A status, with ITR being the best predictive measure (ITR <30 out of 36, indicating nearly 90% chance of being A+). |

Legend: Aß, amyloid ß; A+, amyloid- ß positive; A-, amyloid- ß negative; ACS, Alzheimer’s Clinical Syndrome; aMCIsd, amnestic Mild Cognitive Impairment single domain; aMCImd, amnestic Mild Cognitive Impairment multiple domains; APOE, Apolipoprotein E; CT scan, Computed Tomography scan; ComCBS, cortical syndrome; CDR-SB, Clinical Dementia Rating Scale - Sum of Boxes; CSF, Cerebrospinal Fluid; DCR, Delayed Cued Recall; DFR, Delayed Free Recall; DTR, Delayed Total Recall; fMRI, functional Magnetic Resonance Imaging; FTD, frontotemporal dementia; ICR, Immediate Cued Recall; IFR, Immediate Cued Recall; IR, Immediate Recall; ISC, Index Sensitivity of Cueing; MCI-AD, Mild Cognitive Impairment that progressed to Alzheimer’s Disease; MCI-S, stable Mild Cognitive Impairment; MEG, Magnetoencephalography; MRI, Magnetic Resonance Imaging; MRS, Magnetic Resonance Spectroscopy; MTL, medial temporal lobe; NAA/Cr, *N-acetyl* aspartate/creatine ratios; naMCI, non-amnestic Mild Cognitive Impairment; NGT with 1-h-high, normal glucose tolerance with 1-hour post-load plasma glucose ≥155 mg/dl; NGT 1-hour-low, normal glucose tolerance with 1-hour post-load plasma glucose <155 mg/dl ; PET, Positron Emission Tomography; PSP, progressive supranuclear palsy; p-tau, phosphorylated tau; SCD, subjective cognitive decline; sMRI, structural Magnetic Resonance Imaging, SPECT, Single Photon Emission Computed Tomography; TCR, Total Cued Recall; t-tau, total tau; VBM, Voxel-Based Morphometry.

**Notes: Encoding impairment rate**: the number of words not recalled in IFR 1st trial, those not recalled in neither IFR 1 nor 2, and those never recalled. +**False Recognition**: a positive response to a distractor. **§Forgetting rate**: number of the recalled words on delayed recall minus that on the third trial. ||**Index Sensitivity to Cueing – ISC** = (48 − IFR) / (ITR - IFR). ¶ **Intrusions**: produced words that did not belong to the study list. ** **IR** = Immediate recall right after recognition. + + **Learning curve**: slope of recall across the three trials. …**Long-term retention rate index** = DTR after 20 minutes / DTR after 1 week. ¹**Recall consistency on the free recall**: number of the words recalled consistently from the first to the third trial. ²**Recognition**: correct recognition in yes or no task.
